# Aquatic Plants, *Landoltia punctata*, and *Azolla filiculoides* as Bio-Converters of Wastewater to Biofuel

**DOI:** 10.3390/plants9040437

**Published:** 2020-04-01

**Authors:** Ana F. Miranda, N. Ram Kumar, German Spangenberg, Sanjukta Subudhi, Banwari Lal, Aidyn Mouradov

**Affiliations:** 1 School of Sciences, RMIT University, Bundoora West Campus, Bundoora VIC 3083, Australia; ana.miranda@rmit.edu.au; 2 The Energy and Resources Institute, New Delhi 110 003, India; n.kumar@teri.res.in (N.R.K.); ssubudhi@teri.res.in (S.S.); banwaril@teri.res.in (B.L.); 3 AgriBio, Centre for AgriBioscience, La Trobe University, Bundoora VIC 3083, Australia; German.Spangenberg@agriculture.vic.gov.au; 4 School of Applied Systems Biology, La Trobe University, Bundoora VIC 3086, Australia

**Keywords:** duckweed, hydrogen, phytoremediation, wastewater treatment

## Abstract

The aquatic plants, *Azolla filiculoides*, and *Landoltia punctate*, were used as complementing phytoremediators of wastewater containing high levels of phosphate, which simulates the effluents from textile, dyeing, and laundry detergent industries. Their complementarities are based on differences in capacities to uptake nitrogen and phosphate components from wastewater. Sequential treatment by *L. punctata* followed by *A. filiculoides* led to complete removal of NH_4_, NO_3_, and up to 93% reduction of PO_4_. In experiments where *L. punctata* treatment was followed by fresh *L. punctata*, PO_4_ concentration was reduced by 65%. The toxicity of wastewater assessed by shrimps, *Paratya australiensis*, showed a four-fold reduction of their mortality (LC_50_ value) after treatment. Collected dry biomass was used as an alternative carbon source for heterotrophic marine protists, thraustochytrids, which produced up to 35% dry weight of lipids rich in palmitic acid (50% of total fatty acids), the key fatty acid for biodiesel production. The fermentation of treated *L. punctata* biomass by *Enterobacter cloacae* yielded up to 2.14 mol H_2_/mole of reduced sugar, which is comparable with leading terrestrial feedstocks. *A. filiculoides* and *L. punctata* can be used as a new generation of feedstock, which can treat different types of wastewater and represent renewable and sustainable feedstock for bioenergy production.

## 1. Introduction

Global climate change, population growth, and increasing pollutions led to global shortages in clean water, which triggered an unprecedented search for new renewable and sustainable bioremediation technologies. The search for plant species that can use wastewater as a source of essential nutrients and that can generate biomass that can be further utilized as feedstock for bioenergy production represents one of the most researched areas worldwide, captivating the interest of both the public and scientific communities [1,2,3,4,5,6,7,8]. Most of the known terrestrial plants cannot be used because they cannot grow even in diluted wastewaters. Microalgae have been extensively investigated because of their high bioremediation rates [9,10,11,12]. However, the high cost of harvesting (up to 30% of total cost) is still the major obstacle for the algal biotechnology industry [9,10,11,13].

Aquatic plants, submerged, emerged (rooted), and free-floating species (Figure S1) that often colonize wetlands started attracting attention due to their ability to generate a large amount of biomass, having high bioremediation rates, and being cheap and easy to maintain and harvest [1,2,3,4,5,7,14]. Among these species, free-floating plants have obvious advantages because of their low harvesting cost. The most investigated aquatic species which have been assessed for wastewater treatment include water hyacinth (*Eichhornia crassipes*) [15,16], water lettuce (*Pistia stratiotes*), [17,18,19], water ferns: Salvinia [20,21] and Azolla species [4,6,22,23,24,25,26], and representatives of the *Lemnaceae* or duckweed [27,28,29,30].

### 1.1. Duckweed and Azolla as Phytoremediation Species

Duckweed species, *Lemna minor*, *Lemna gibba*, *Spirodela polyrhiza* and *Wolffia arrhiza*, have been extensively studied for over 20 years for phytoremediation of contaminated waters [6,31,32,33,34,35,36]. The *Landoltia* genus was recently segregated from the genus *Spirodela* and has attracted attention because of its high growth rate, bioremediation capacity, and chemical composition [37,38,39]. *Landoltia punctata (L. punctata)*, the most investigated representative of the *Landoltia* genus, showed high phytoremediation capacities along with high growth rates with an annual yield up to 39.2–55 t dw/ha-yr, which is higher than yields of the main bioenergy plants, such as switchgrass (5.2–26 t dw/ha-yr) and miscanthus (5.0–44 t dw/ha-yr) and woody plants, such as poplar (9–15 t dw/ha-yr) [4,16,34,40,41,42,43]. Azolla (also known as mosquito fern, duckweed fern, fairy moss, and water fern) has become increasingly popular because of its biomass production and bioremediation potential [4,6,22,23,24,25,26,44]. Unlike most of the terrestrial and aquatic plants, Azolla can grow efficiently even in the absence of nitrogen in media utilizing the nitrogen-fixing capacity of its natural symbiont, the endophytic cyanobacterium, *Anabaena azollae* Strasburger (*A. azollae*) [45,46,47]. This symbiosis is associated with the fixation of up to 1.1 t/ha-yr of nitrogen, which is significantly higher than the nitrogen fixation rate of legumes (0.4 t N/ha-yr) [23,48,49]. Doubling its biomass every 2–7 days, Azolla is one of the world’s fastest growing plants, with productivity varying between 2.9 and 5.8 g dw/m^2^-day (10.5–21.1 t dw/ha-yr) when grown on artificial media, wastewaters and maturation ponds [49]. Under optimal conditions in natural ecosystems, such as rivers, lagoons and irrigation channels, Azolla can bloom with growth rates up to 300 g/m^2^-day of fresh biomass (1095 t/ha-yr) [50] and 25.6–27.4 g dw/m^2^-day of dry biomass (93.4–100 t dw/ha-yr) [4,23,51]. Growth in wastewater is associated with the removal of total nitrogen (TN) and total phosphorus (TP) with rates of up to 2.6 t N/ha-year and 0.434 t P/ha-yr, respectively [6,22,23,52].

### 1.2. Duckweed and Azolla as Universal Feedstock for Biofuel Production

Duckweed species have been extensively used as feedstocks for bioethanol production over the last 20 years [6,53,54]. Azolla is a new bioenergy feedstock, and its promising potential is based on its unique chemical composition [4,5,6,25,55]. Together with their evolutional symbiont, *A. azollae*, Azolla representatives contain three major types of energy molecules, lignocellulose, sugars/starches, and neutral lipids, which are found separately in known terrestrial feedstocks and microalgae [4,6,22,23,25,51,52]. *A. filiculoides*, *L. punctata*, and *L. minor* species were earlier used as feedstocks for two thermochemical technologies, pyrolysis and thermochemical liquefaction [4,6,30,53,56]. 

Hydrogen (H_2_) is a valuable source of clean energy and is a feedstock for some industries for which demand has increased considerably in recent years. Terrestrial crops that are rich in carbohydrates, such as cellulose and starch, were widely used for hydrogen production. Aquatic plants are attracting attention as feedstock for biohydrogen production because of their biomass yield and biochemical contents [57]. *A. filiculoides* could produce up to 2.43 mol H_2_/mole of reduced sugar, which is comparable with the H_2_ production obtained from leading feedstocks [4,58].

In a previous study, the sequential treatment of swine wastewater, which is significantly higher in TN than TP (TN>>TP) by *L. punctata* and *A. filiculoides*, had a strong complementing and additive effect which was not significantly changed by seasonal changes in temperature and photoperiod [6]. However, some wastewaters, like those resulting from the textile dyeing, finishing, and laundry detergent industries, are higher in TP than TN.

This study aims to assess the additive and complementary phytoremediation capacities of *L. punctata* and *A. filiculoides* plants for the treatment of wastewater, which is significantly higher in TP than TN. The complementarities of these plants in the treatment of TP>>TN type of wastewaters were assessed based on differences of their capacities to uptake these nutrients, particularly on the ability of Azolla to uptake TP in the absence of TN. To increase the toxicity of wastewater, selenium (SeO_2_) was added to a concentration of 0.8 mg/L (0.6 mg/L of Se), which mimics typical Se concentration found in landfill leachates [59,60,61]. Acute toxicity tests of treated wastewater using the freshwater shrimp, *Paratya australiensis*, showed that the sequential treatment by *L. punctata* and *A. filiculoides* significantly reduced their mortality, increasing the LC_50_ value four-fold. Because of the high carbohydrates content (high C/N ratio) of both species’ biomass, they were used (i) as an alternative carbon source for heterotrophic marine protists, thraustochytrids, which produced up to 35% DW of lipids high in palmitic acid (C16:0, 50% of fatty acid methyl esters, FAME), which is a valuable feedstock for biodiesel production and (ii) as a feedstock for bio-hydrogen production via fermentation by *Enterobacter cloacae (E. cloacae)*.

## 2. Results and Discussion

### 2.1. Wastewater Treatment with L. punctata and A. filiculoides 

#### 2.1.1. Removal of Nitrogen and Phosphorus by *L. punctata*

**Figure 1:**
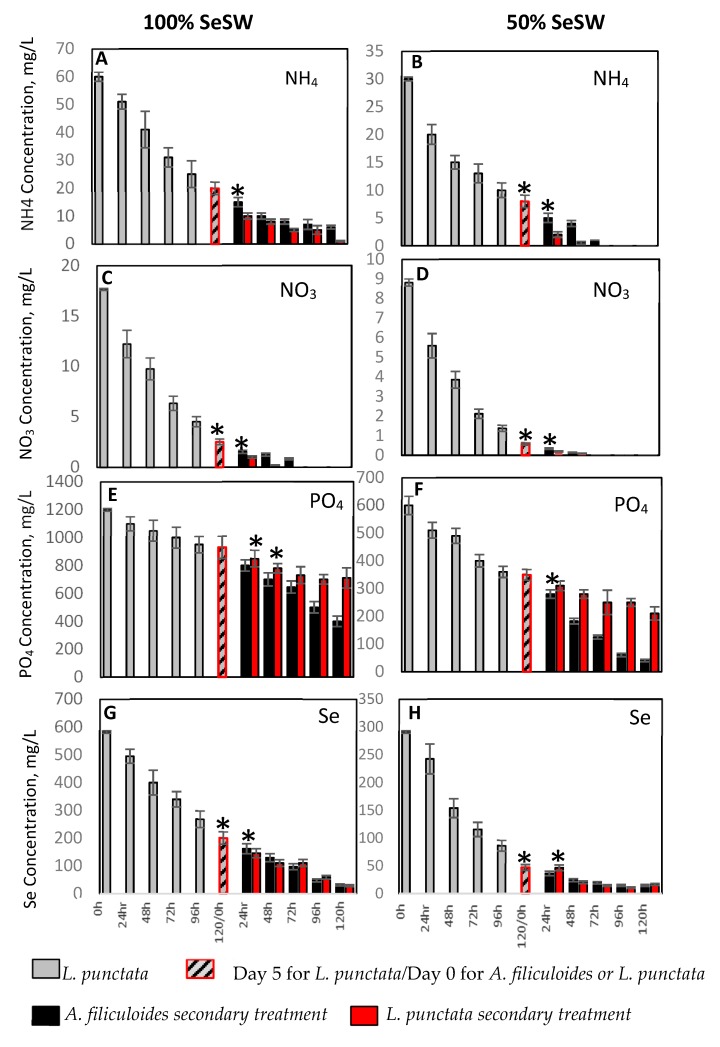
Reductions of concentrations of PO_4_, NH_4_, NO_3_ and Se in 100% (**A**,**C**,**E**,**G**) and 50% (**B**,**D**,**F**,**H**) SeSW by *L. punctata* followed by sequential treatment with *A. filiculoides* or *L. punctata*. Data expressed as mean ± standard deviation, * Significance levels: *p* < 0.05.

In the phytoremediation experiments, we used selenium-containing synthetic wastewater (SeSW, Table S1), which is low in TN (70 mg/L), high in TP (1.2 g/L) and contained 0.8 mg SeO_2_/L. Both 100% and 50% SeSW were initially treated for five days by *L. punctata*, followed by an additional period of five days of treatment by *A. filiculoides* (Figure S2). Treatment of 100% SeSW with *L. punctata* led to a decrease in NH_4_ concentration from 60.0 mg/L to 20.7 mg/L (67% uptake) (Table 1, Figure 1). The concentration of NO_3_ decreased more dramatically, showing 87% uptake over the five days of the experiment. Because of a very high concentration of PO_4_ (1.2 g/L), five days of treatment led to just 23% uptake with a reduction to a concentration of 931 mg/L. Diluted (50%) SeSW was less stressful to *L. punctata*, which was reflected by higher rates of nutrient uptake: 74%, 43%, and 94% for NH_4_, PO_4_ and NO_3_, respectively (Table 1, Figure 1). A broad spectrum of absorption rates of nutrients by *L. punctata* and other duckweed species has been reported [6,14,30,32,34,35,36,58,62,63,64,65,66,67,68].

Short-term treatment of the SeSW by *L. punctata* did not lead to a significant increase in biomass production (1.5-fold, Table 1). In general, the growth of the aquatic plants on wastewaters increase exponentially after a lag phase observed over the first 3–5 days as a result of adaptation to the new growing environment [4,33]. This period, however, is associated with the intensive absorption of the key nutrients from wastewaters, which normally leads to strong exponential growth after the lag phase [4,33,69]. For example, lack of growth of *Spirodela punctata* (duckweed) in synthetic wastewater over the first 100 h was associated with absorption rates of 11.5 mg NH_4_/L-day and 0.9 mg PO_4_/L-day [33]. This was followed by the fast-growing period with uptake rates of 22.9 mg NH_4_/L-day and 3.1 mg PO_4_/L-day.

#### 2.1.2. Secondary Treatment of SeSW by *A. filiculoides*

After five days, *L. punctata* plants were removed, the treated SeSW was filtered, and fresh *A. filiculoides* plants were added in amounts enough to cover the surface of the plastic containers (Figure S2). Five additional days of treatment of 100% SeSW with *A. filiculoides* led to a further reduction in the concentration of all three nutrients, NH_4_, PO_4_ and NO_3_ to 6.1 mg/L (70.4% uptake), 404.2 mg/L (57% uptake) and 0 mg/L (100% uptake), respectively (Figure 1, Table 1). Total reductions of these three nutrients from 100% SeSW after ten days of treatment by *L. punctata* and *A. filiculoides* were 90%, 67% and 100% for NH_4_, PO_4,_ and NO_3_^,^ respectively. The secondary treatment of 50% SeSW by *A. filiculoides* showed complete removal of NH_4_ and NO_3_ and reduction of PO_4_ to 40.5 mg/L (89% reduction). The total reduction of these three nutrients from 50% SeSW after ten days of treatment with *L. punctata* and *A. filiculoides* were 100%, 93% and 100% for NH_4_, PO_4,_ and NO_3_, respectively.

In control experiments, fresh *L. punctata* biomass was added instead of *A. filiculoides* after the first five days (Figure S2). For 100% SeSW, this treatment was associated with a 96% reduction of NH_4_ and complete removal of NO_3_. The concentration of PO_4_ was, however, reduced at a much lower rate than treatment by *A. filiculoides*. The final concentrations of PO_4_ were reduced to 710 mg/L and 210 mg/L for 100% and 50% SeSW, 24% and 41%, respectively (Figure 1, Table 1). Total reductions of PO_4_ after ten days of treatment with *L. punctata* and *L. punctata* were 43% and 65%, from 100% and 50% SeSW, respectively.

The differences in absorption of TN and TP by plants reflect their significance in plant metabolism, biochemistry, and growth. For terrestrial and aquatic plants grown in their natural ecosystems, the absorption ratio of TN:TP is between 5:1 and 16:1, which correlates with average uptake rates of these nutrients: 45–1670 mg TN/m2-day and 8–220 mg TP/m2-day [22,70,71,72,73,74]. Grown in TN-rich wastewaters, the typical nutrient content of aquatic plant biomass can be 22–63 gTN/kg dw and 3–14 gTP/kg dw [35,75,76,77]. Reduction in the concentration of TN in treated wastewater limits aquatic plant growth, which, in turn, reduces TP uptake [22,33]. As a result, TP is the main wastewater contaminant after phytoremediation-mediated treatment, which is more obvious for wastewaters that are higher in TP than TN, such as municipal wastewaters and industrial, textile dyeing and finishing and laundry detergent industries. The additional treatments of wastewater with fresh aquatic plant species, such as *L. punctata*, shows no significant reduction in TP since TN is a limiting factor. The unique ability of Azolla/*A. azollae* association to absorb TP even in absence of TN makes it an ideal secondary phytoremediation agent, which was shown in our experiments.

#### 2.1.3. Removal of Selenium from by Sequential Treatment with *L. punctata* and *A. filiculoides*

In the aquatic environment, Se exists in the forms of two soluble molecules, selenate (SeO_4_) and selenite (SeO_3_), and both of these molecules are of major concern because of their toxicity and stable bioaccumulation in different tissues [78,79,80]. Concentrations of Se in wastewaters can vary significantly. Landfill leachates can contain up to 590 µg Se/L, which is up to 3000-fold higher than its concentration in freshwater [5,59,81,82,83]. Phytoremediation was widely used for the absorption of Se from contaminated waters [60,61].

In our study, treatment of SeSW with *L. punctata* led to 65% uptake of Se from 100% SeSW and 84% from 50% SeSW (Figure 1, Table 1). This absorption was associated with an accumulation of 0.56 mg Se /g DW and 0.26 mg Se/g DW, from 100% and 50% SeSW, respectively, in the plant’s biomass (Table 2). Secondary treatment with *A. filiculoides* led to markedly greater, 94.6% and 95%, removal of Se from 100% SeSW and 50% SeSW, respectively, with the accumulation of 0.42 mg Se/g DW and 0.18 mg Se/gDW in their biomass, respectively (Table 1 and Table 2). *A. filiculoides* showed an approximately similar absorption rate of Se in our previous experiments [4]. *Azolla caroliniana*, after 14 days of treatment, showed absorption rates of 1 mg Se/g DW from 2.5 mg/L Se solution [84]. Lower absorption rates were observed for *Salvinia rotundifolia* (0.7 mg Se/g dw), *Lemna minor* (500 mg Se/g dw) and *Eichhornia* (300 mg Se/g dw) [84]. Sequential treatment with *L. punctata* showed a similar Se absorption profile to *A. filiculoides*, shown in Figure 1, Table 1 and Table 2.

#### 2.1.4. Shrimp Toxicity Tests of Treated Wastewaters

**Figure 2:**
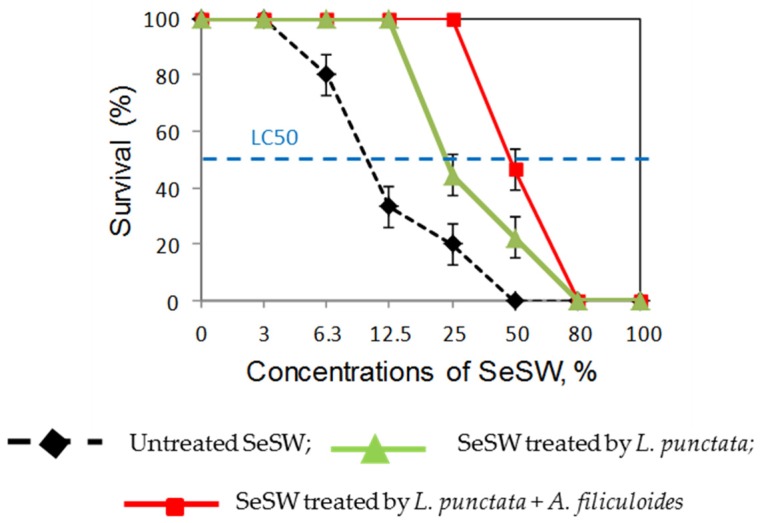
Survival rates of *P. australiensis* in untreated and treated by *L. punctata* and *A. filiculoides* SeSW.

We used freshwater shrimps, *Paratya australiensis*, as biosensors of the treated wastewater’s quality. In our control experiments, we assessed the acute toxicity of SeSW on freshwater shrimps, *P. australiensis*, that were exposed to different dilutions of SeSW (0%, 3%, 6.3%, 12.5%, 25%, 50%, 80% and 100%) (Figure 2). Shrimp survival was 100% in very diluted, 3%, SeSW. However, at concentrations higher than 50% SeSW, all shrimps were found dead after four days (100% mortality). The LC_50_ of untreated SeSW was found to be 11.22%.

The same dilutions of SeSW collected after treatment by *L. puntacta* showed reduced acute toxicity with 100% mortality observed at concentrations ≥ 80% (LC_50_ = 27.80%). Secondary treatment of SeSW with *A*. *filiculoides* significantly reduced shrimp’ mortality, increasing the LC_50_ value four-fold (46.9% compared to untreated SeSW). As a result, a concentration of SeSW ≤ 25% showed zero toxicity to the population of *P. australiensis* after treatment with both plants.

Azolla can be severely affected in even much-diluted swine water effluents [4,6,22]. Moreover, high concentrations of TN can reduce the nitrogen-fixing activity of *A. azollae* [22]. This indicates that the initial treatment of the wastewaters by duckweed can be beneficial for Azolla. This suggests that duckweed and Azolla can have additive and complementary effects in the treatment of different types of wastewaters.

#### 2.1.5. Suppression of Microalgal Growth by *L. punctata* and *A. filiculoides*

The combination of TN, TP, nutrients and sunlight often leads to microalgal and cyanobacterial blooms, which are highly undesirable contaminants in the wastewater effluents. The removal of these microorganisms makes the water treatment process more expensive. The abilities of free-floating aquatic species, both duckweed, and Azolla, to create dense mats on the surface of the water can have the potential to suppress microalgal/cyanobacterial cells’ growth through reduction of (i) the light penetration beneath the plants and (ii) the concentrations of the main nutrients. To quantify the effect of duckweed and Azolla on algal growth, we grew common microalgae found in wastewaters, *Chlorella vulgaris*, for three weeks in growth media covered by mats of duckweed and Azolla. Figure S3 shows extensive growth of *C. vulgaris* in the control experiment, with an 8.6-fold increase in cell concentrations at the end of the experiment. Very little *C. vulgaris* growth (2.1 × 10^4^ cells/mL cells) was detected in the containers covered by the Azolla plants. Containers covered by growing duckweed showed intermediate growth rates of *C. vulgaris*. Measurements of the light penetration to the bottom of the containers showed the lowest light intensities for the containers covered by Azolla. Analysis of the N and P in media showed that the greatest uptakes of both key nutrients were observed in the duckweed experiment, which can be explained by the additive effects of the duckweed and viable *C. vulgaris* cells (Figure S3). These experiments show the additional advantage of duckweed and Azolla-based phytoremediation which completely suppresses algal growth in treated wastewater effluent.

### 2.2. Biofuel Production from L. punctata and A. filiculoides

#### 2.2.1. *L. punctata* and *A. filiculoides* as an Alternative Carbon Source for Lipid Production

Application of duckweed and Azolla representatives for biodiesel production is not economical because of low lipid yields (5–11% DW for *L. punctata* and 6–8% DW for *A. filiculoides* and *A. pinnata*) [25,30,55,83,85]. However, high growth rates, short rotation time, along with high carbohydrate content makes these aquatic plants a potential feedstock for oleaginous heterotrophic microorganisms. Duckweed carbohydrates mainly contain cellulose/hemicellulose (17.6–51% DW) and starch (21–38% DW) [30,84,86,87]. Together with their evolutional symbiont, *A. azollae*, Azolla representatives contain 6% DW of starch and up to 35% DW of cellulose/hemicellulose [4,22,23,25,52]. Biochemical assessment of *L. punctata* and *A. filiculoides* showed that after wastewater treatment they accumulate up to 22% DW of total carbohydrates (Figure S4).

**Figure 3:**
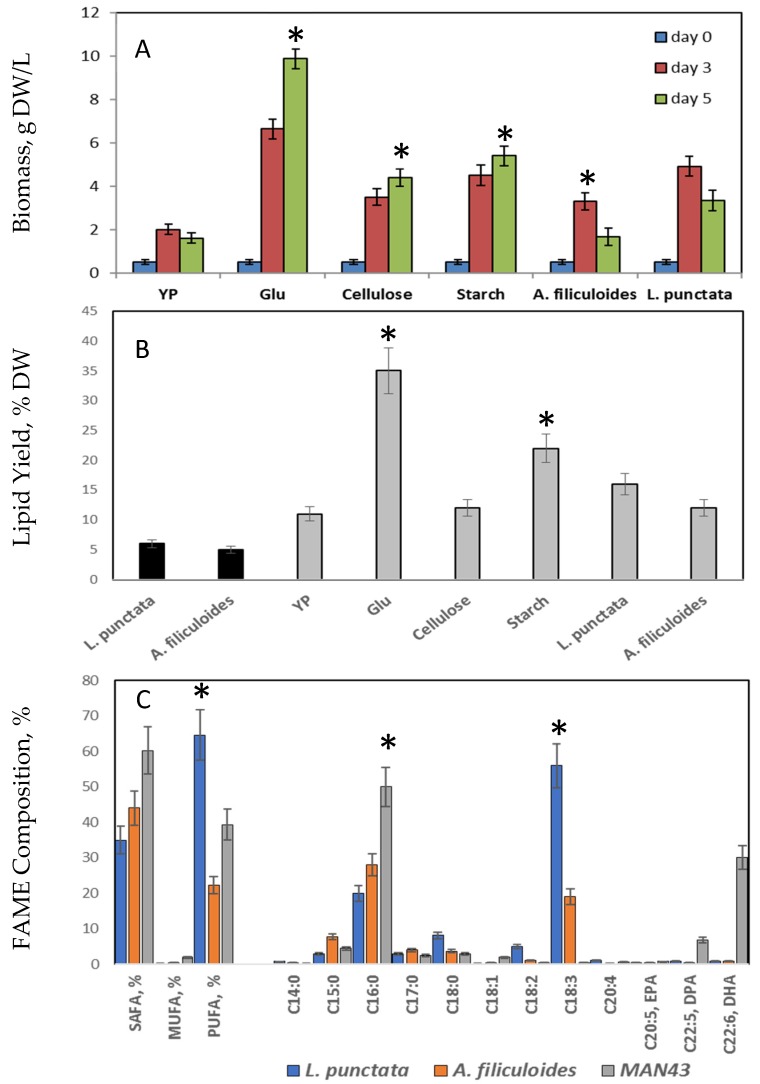
(**A**): Growth of MAN43 cells in YP media supplemented with different carbon sources. Glu: glucose; (**B**): Lipid yields in *L. punctata*, *A. filiculoides* and MAN43 cells grown on different carbon sources. Black boxes: total lipids extracted from *L. punctata* and *A. filiculoides*; grey boxes: lipid yields in MAN43 cells grown on different carbon sources. Glu: glucose; Gly: glycerol. (**C**): FAME composition of lipids extracted from *L. punctata, A. filiculoides* and MAN43 grown on glucose. * Significance levels: *p* < 0.05.

Unicellular, heterotrophic marine protists, thraustochytrids, have been extensively studied over the last decade because of their biotechnological applications in human health and biodiesel production [88,89,90,91,92]. They are rich in lipids (up to 50% DW) and are known for their ability to grow on dead mangrove leaves secreting cellulose- and starch-degrading enzymes [93,94,95]. A new thraustochytrid strain, MAN43, isolated from mangrove sediments was assessed in this study for its growth and lipid production using on *L. punctata* and *A. filiculoides* biomass as a carbon source (Figure 3 and Figure S5).

**Figure 4:**
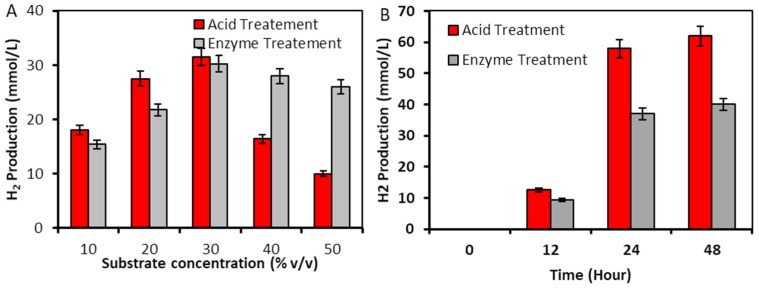
(**A**) Hydrogen production by E. cloacae DT-1 from different concentrations of acid-treated prehydrolysate and enzymatically saccharified duckweed biomass; (**B**) hydrogen production performance of *E. cloacae* DT-1 from optimum concentrations of acid-treated prehydrolysate and enzymatically saccharified hydrolysate of duckweed biomass, under decreased partial pressure.

The biomass production of MAN43 grown on treated dry *L. punctata* and *A. filiculoides* biomass was compared with its growth rates on YP media and YP supplemented with reducing sugar, glucose, and starch and cellulose, the main components of carbon polymers in duckweed and Azolla [5] (Figure 4 and Figure S6). MAN43 showed a high growth rate (9.8 g/L) after five days of glucose. The use of starch and cellulose showed lower biomass production than glucose, but significantly higher than on YP alone (*p* ≥ 0.05). Growth on cellulose and starch was correlated with the activities of corresponding degrading enzymes, amylase, and cellulase secreted by MAN43 cells (Table 3). The amylase activity after three days of growth on starch was increased three-fold (from 10.2 to 29.3 units min^−1^ ml^−1^). The activity of cellulase enzymes was also increased in the presence of cellulose: from 2.0 to 18.1 units min^−1^ ml^−1^ (9-fold). MAN43 grown on treated *L. punctata* and *A. filiculoides* showed biomass production up to 2.4-fold higher than on YP alone. This growth was also correlated with increased amylase and cellulase activities in the supernatant. 

When grown on glucose, MAN43 cells contain 35% DW of lipids, with FAMEs mainly represented by palmitic acid (C16:0, 50% of FAMEs) and DHA (31% of FAMEs), the key fatty acids for biodiesel and food supplements production (Figure 3). The MAN43 lipid level and composition are different from the same of *L. punctata* and *A. filiculoides*, which accumulate 6% DW and 5% DW, respectively. FAMEs composition of *L. punctata* was mainly represented by C16:0 (palmitic acid, 20% of FAME) and C18:3 (linolenic acid, 56% of FAME), comprising together 76% of total FAME, which is similar to previously published data [55,85]. FAME composition of A. *filiculoides* is mainly represented by palmitic acid, 21% of FAME and by linolenic acid (19% of FAMEs). This data showed that, collected after wastewater treatment, *L. punctata* and *A. filiculoides* biomass can produce up to three times as many lipids and better FAME composition for biodiesel production if they will be used as a carbon source for lipid-rich heterotrophic microbes.

#### 2.2.2. Hydrogen Production from *L. punctata*

The chemical composition of duckweed representatives, such as a high concentration of starch and the absence of lignin, led to intensive research on their application for biofuel production [30,83,86]. Analysis of starch concentration in the dry *L. punctata* biomass grown on ½ Hoagland nutrient media showed 17.2 ± 3.4% DW, which is comparable with an average starch yield in duckweed species [30,82,83,85,86,96,97]. We used pretreated dry biomass of *L. punctata* for the production of H_2_ using *E. cloacae* as a fermentation inoculum. Two treatment methods were applied for the production of reduced sugars: acid pretreatment with 1% sulphuric acid in an autoclave for 60 min at 121 °C, and enzymatic saccharification of a pretreated solid biomass fraction (released after acid treatment) with Cellic CTec2 (cellulase complex, Novozyme). Acid treatment led to the production of glucose (12 g/L), arabinose (0.04 g/L), xylose (4.36 g/L), as the main sugar monomers. The enzymatically saccharified hydrolysate was composed mainly of glucose and xylose, 11.8 g/L and 0.92 g/L, respectively, with glucose comprising more than 80% of the total reduced sugars (Table 4). Treatment with Cellic CTec2 yielded higher glucose content compared to pectinase treatment previously reported by Chen, et al. [98].

For optimization of substrate concentrations, acid-treated prehydrolysate and enzymatically saccharified hydrolysate was diluted in the following proportions (*v/v*): 10%, 20%, 30%, 40% & 50% with the BSH medium. The *E. cloacae* DT-1 strain showed growth and hydrogen production from all the concentrations of substrates. Maximum hydrogen yields were observed from 30% substrates: 32.48 mmol/L and 30.22 mmol/L for acid- and enzymatically treated prehydrolysate, respectively (Figure 4). The acid-treated prehydrolysate, at 30% dilution, contained 5.02 g/L of total reduced sugar and enzymatically treated prehydrolysate, at 30% dilution, contained 3.8 g/L of total reduced sugar. The H_2_ yields in our experiments were up to six-fold higher compared to the H_2_ yield obtained after fermentation of the 30 g/L acid-treated duckweed reported earlier by Aslan [57]. For all the other substrate concentrations, higher H_2_ yields were observed for the acid-treated prehydrolysates, which can be explained by their higher concentrations of reducing sugars (Table 5). An increase in substrate concentration led to much a sharper decrease in H_2_ production from acid-treated prehydrolysates than enzymatically treated hydrolysates. This can be explained by the higher production of ethanol and short-chain organic acid as co-products during hydrogen production [98,99,100,101]. Further studies were performed by using a 30% concentration of substrates. 

The high partial pressure of hydrogen (pH_2_) triggers the production of ethanol and lactate instead of H_2_, acetates and butyrate [101]. To avoid this, we conducted batch fermentative hydrogen production experiments under decreased pH_2_. This was achieved by reducing the total pressure of biogas in the headspace of the fermenter. The performance of the one-liter scale batch fermenter system was evaluated regarding volumetric H_2_ production. As a result, 62 mmol/L of H_2_ was obtained from acid-treated prehydrolysate with a H_2_ yield efficiency of 2.14 mol H_2_/mole of reduced sugar (Table 5). This was associated with a final pH decrease from 7.5 to 5.42. The volumetric hydrogen production increased two-fold under decreased partial pressure compared to the previous batch experiment. Fermentation of the enzymatically saccharified hydrolysate under decreased pH_2_ led to the production of 40 mmol/L of H_2_. This was associated with a pH decrease to 5.64. The resulting volumetric yield efficiency was 1.8 mol H_2_/mol of reduced sugar, which is lower than from acid-treated prehydrolysate (Table 5). The higher yield efficiency for acid-treated hydrolysate could be attributed to the presence of higher C5 sugar concentrations. In both cases, the hydrogen production rates increased exponentially between 12 and 24 h followed by the stationary phase at 24 h, which can be attributed to the depletion of sugar and production of metabolites such as acetic acid and ethanol and a decrease of pH. The experiments were terminated after 48 h. Terrestrial plants have been widely explored for use as a feedstock for biohydrogen production [98,102,103,104,105,106] (Table S2).

Hydrogen yield efficiencies reported from these feedstocks are in the range of 0.44 to 2.84 mol H_2_/mole of the substrate. Aquatic plants have not been much explored as feedstocks for biohydrogen production. Previously, Aslan [57] and Xu and Deshusses [58] reported production of 10.71 and 3.34 mmol hydrogen/L, respectively, from acid-treated duckweed biomass. In comparison, the hydrogen production performance of *E. cloacae* DT-1 strain from *L. punctata* biomass reported in this study is significantly higher. This also reveals a high potential of *E. cloacae* DT-1 strain for the conversion of reduced sugars into H_2_. This strain showed potential for the utilization of a broad-spectrum of sugars. Earlier we showed that pre-treated *A. filiculoides* biomass could produce 2.43 mol H_2_/mole of reduced sugar after fermentation with *E. cloacae* DT-1 [4]. The higher H_2_ yield from *A. filiculoides* could be due to different sugar concentration and composition of the different feedstock and the production of different fermentative by-products. The production of butyric acid as a co-metabolite results in less hydrogen production. H_2_ production from *L. punctata* biomass by DT-1 resulted in a comparatively higher concentration of butyric acid and thus resulted in less hydrogen production (Table 5).

## 3. Materials and Methods 

### 3.1. Growth of Azolla and Duckweed 

Both *A. filiculoides* and *L. punctata* were obtained from the collection of aquatic plants at RMIT University. The plants were collected and rinsed with deionized water to remove algae and other debris. Experiments were set up in plastic containers containing 500 mL of 100% or 50% of SeSW (Table S1). Details of the experimental design are shown in Figure S2. To each container, the same amount of fresh plants and enough to cover the entire water surface with a single layer of fronds was placed. The containers were then placed in a 23 °C growth chamber with a 16 h photoperiod and a photosynthetic photon flux density of 50 μmol/m^2^/s. The test solution in each container was mixed daily. After five days of the experiment, all *L. punctata* plants were removed and new plants, *A. filiculoides* or *L. punctata*, were added, starting an additional period of five days of treatment. Three replicates were included for each treatment. Every day over five days, water samples and biomass were collected to evaluate the levels of Se, ammonia, nitrate and phosphate. Concentrations of cations and anions were measured using the ion chromatography system Dionex ICS-1100 (Thermo Scientific, Waltham, MA, USA). The biomass dry weights were determined immediately after sampling by drying samples at 80 °C overnight. Biomass production measured as the dry weight was calculated according to [5].

### 3.2. Selenium Extraction and Measurements

Liquid SeSW samples were filtered through a 0.45 µm syringe filter and acidified with concentrated HNO_3_ to 2% and kept at 4 °C. Plants from each treatment were rinsed twice with Milli-Q water, blotted on filter paper and dried at 70 °C overnight. Dried samples were then ground using a mortar and pestle and a sub-sample of 100 mg was weighed into glass tubes and digested with HNO_3_ (68.5%): HClO_4_ (70%) mixture (1 mL, 10:1, *v/v*) in a dry heating block at 100 °C for 30 min [106]. After cooling to room temperature, samples were filtered using a 0.45 µm syringe filter and diluted to 10 mL with Milli-Q water. Plant extracts and wastewater were analyzed for total selenium concentration by inductively coupled plasma mass spectrometry (ICP-MS) (Model 4500 series 300, Agilent Technologies, Santa Clara, CA, USA).

### 3.3. Shrimp Toxicity Test

To assess the effectiveness of the remediation by aquatic plants, toxicity tests were performed using a common Australian test species: the freshwater shrimp, *Paratya australiensis*. This species was obtained from RMIT’s collection and was maintained in 20 L glass tanks containing dechlorinated filtered water at 24 °C, with a 16:8 h light: dark photoperiod. Shrimps were fed daily with algae wafers and trout pellets except for 24 h before the commencement and during the toxicity tests. The toxicity tests were performed under static conditions in 500 mL glass beakers for 96 hours. Each glass beaker contained five shrimps (10–15 mm long) and there were three beakers for each concentration. The LC_50_ was calculated for each treatment using probit analysis, maximum likelihood method. All the statistical analysis was performed using a ToxRat 3 Software (ToxRat Solutions GmbH).

### 3.4. Thraustochytrids Isolation and Growth

Thraustochytrids cells were collected from mangrove sediments (Victoria, Australia. GPS location: -38.265605 144.496448). The collected samples were vortexed for five seconds to resuspend the microorganisms present and plated on GPY media (10 g/L Tryptone, 5 g/L Yeast extract, 30 g/L glucose in 50% seawater) containing a cocktail of antibiotics (Penicillin 50 µg/ml, Nystatin 10 µg/ml, Rifampicin 50 µg/ml, Streptomycin 50 µg/ml) according to Gupta, et al. [107]. Isolated strains were identified phylogenetically using a maximum-parsimony and distance trees generated by using partial 18S rRNAs genes amplified with a set of primers for fungi/yeast described in Burja, et al. [108]. The 18S rRNA gene sequences were compared with those of the other strains obtained from GenBank at the National Center NCBI (http://www.ncbi.nlm.nih.gov/). Multiple sequence alignments were conducted with the CLUSTAL W at http://www.ebi.ac.uk/clustalw/index.html. MEGA7 (Molecular Evolutionary Genetics Analysis version 7.0) was used to generate maximum-parsimony and distance trees. Identified *Thraustochytrium* strain was designated as MAN43 (Genbank accession numbers: MH790120). 

### 3.5. Nile Red Staining

For Nile Red staining the thraustochytrids cells were incubated in 1 mL of 20% DMSO containing 5 μL of Nile Red stock solution (0.10 mg/mL of Nile Red dissolved in acetone) for 5 min. The stained samples were then subjected to fluorescent microscopy analysis to observe the formation of lipid droplets using a Leica DM 2500 microscope with an attached camera Leica DFC 310 FX. Nile-Red filter: excitation at 543 nm, emission 555–650 nm.

### 3.6. Lipid Extraction and Fatty Acid Composition 

Lipid extraction and fatty acid composition were analysed according to Miranda et al. [55]. Briefly, around 25 mg of freeze-dried and ground material was extracted in 4 mL of chloroform/methanol (2:1, *v*:*v*) overnight. After centrifugation, the supernatant was transferred to a pre-weighed 5-mL glass vial and the organic phase was removed under a stream of nitrogen. The content of total crude lipids in each sample was determined by weight difference. To determine the fatty acid composition of the lipids, 2.4 mL of 6% H_2_SO_4_ in methanol was added to each vial, and the vial was sealed with a Teflon cap. Fatty acids were methylated by acid-catalysed transesterification at 80 °C for 3 h. After cooling to room temperature, formed fatty acid methyl esters (FAMEs) were extracted into hexane containing an internal standard (octacosane, 15 mg/L) and analysed directly by GC-MS. The separation of the FAMEs was achieved by a BPX-70 column (50 m × 0.22 mm ID, 0.25 μm film thickness, Trajan Scientific, VIC 3134 Australia) with a constant flow of 1.0 mL/min helium as carrier gas and the following oven temperature program: 120 °C to 245 °C ramping at 3 °C/min, with a total run time of 42 min. The detection was by an Agilent 7000 GC/MS Triple Quad (Agilent, Santa Clara, CA, USA) with the following settings: scanning mass range of 40–550 amu, transfer line temperature of 240 °C, source temperature of 280 °C, the quad temperature of 150 °C and a solvent delay of 4.1 min. A standard mix (C4-C24, Supelco, Sigma-Aldrich Pty Ltd, Castle Hill NSW 1765 Australia) containing 37 FAMEs was used to provide absolute quantification of each fatty acid methyl ester.

### 3.7. Biohydrogen Production

#### 3.7.1. Microorganism, Media and Growth Condition

Isolation and growth conditions of *Enterobacter cloacae* (*E. cloacae*) DT-1 (Gene Bank accession number: JX885522), acid pretreatment and enzymatic saccharification of pre-treated of *L. punctata* was described in Miranda et al. [4].

#### 3.7.2. Batch Dark Fermentation Experiments 

Batch dark fermentation experiments were conducted as previously described [4]. Laboratory-scale batch fermentative hydrogen production was conducted using 2 L batch reactors containing 160 mL of anaerobically prepared BSH medium supplemented separately with acid-treated prehydrolysate (50 % *v/v*, 11 g/L of reducing sugars) and enzymatically hydrolyzed sugars (33 % *v/v*, 4.8 g/L reducing sugar) as feedstock. The initial pH of the media was maintained at 7.5, and 10 % (*v/v*) freshly grown DT-1 culture was used as inoculum. The bottles were incubated at 37 °C for 72 h under static conditions. The generated biogas was collected under decreased partial pressure of H2 (by reducing the total pressure of biogas in the head space of the fermenter). This entailed using a water displacement system involving an inverted water-filled bottle to exert pressure on the head space of the fermenter which allowed displacement of the biogas immediately after its generation within the batch fermenter. The biogas production was monitored by measuring the displaced water collected in a graduated inverted water displacement system containing saline solution at ambient temperature. All experiments were performed in duplicate. Qualitative detection of hydrogen was done by gas chromatography.

#### 3.7.3. Analytical Methods

Bacterial growth was detected by measuring the optical density at 600 nm in a spectrophotometer. The biogas composition generated in the headspace and the soluble metabolites generated during the dark fermentation process were detected by gas chromatography by following the protocols reported by [100]. A High-Performance Liquid Chromatograph (HPLC, Agilent LC 1200 series, USA) equipped with HI-Plex H+ column (Agilent, USA) and 2,5 dinitro- salicylic acid method was used for the quantification of sugars [109]. Sulphuric acid (0.005 N) was used as the mobile phase at a flow rate of 0.55 mL/min. The soluble metabolites were determined using a gas chromatograph (7890A, Agilent, USA) equipped with a flame ionization detector and DB-WAXetr column (60 m × 320 µm × 1 µm). The oven temperature was ramped at 20 °C/min up to 110 °C and 70 °C/min up to 160 °C and then held at 160 °C. The injector and detector temperatures were maintained at 250 °C and 300 °C, respectively. Nitrogen was used as a carrier gas. Starch was analyzed by Starch (HK) Assay Kit (Sigma). All analyses were conducted in duplicate.

### 3.8. Statistical Analysis

All experiments in this study were conducted at least in triplicate. All data are expressed as a mean ± standard deviation. Data variance and normality were assessed by Levene’s and Kolmogorov-Smirnov tests, respectively. The experimental data were subjected to the one-way analysis of variance (ANOVA), as implemented in the Sigmaplot 11 software. Holm–Sidak simultaneous tests were conducted to determine the statistical differences between treatments. To ascertain that the observed variations were statistically significant, the probability *p*-values were determined. A 95% confidence level (*p* < 0.05) was applied for all analyses.

## Figures and Tables

**Table 1:** Nutrients removal from SeSW by *L. punctata* followed by *A. filiculoides* or *L. punctata*.

Species	Biomass, gDW/L		NH_4_ Uptake		PO_4_ Uptake		NO_3_ Uptake		Se Uptake	
	Initial	Final	NH_4_ *, %	NH_4_ ** Total, %	PO_4_-P *, %	PO_4_-P ** Total, %	NO_3_ *, %	NO_3_ ** Total, %	Se *, %	Se ** total, %
**100% SeSW**										
***L. p ^#^***	0.44 ± 0.1	0.66 ± 0.1	66.7 ± 9	NA	22.5 ± 8.8	NA	86.7 ± 11.7	NA	65 ± 9.2	NA
***A. f ^##^***	0.37 ± 0.1	0.5 ± 0.1	70.4 ± 11.2	90 ± 7.9	57.0 ± 11.3	67.0 ± 11.3	100	100	85.1 ± 7.3	94 ± 12.2
***L. p ^##^***	0.32 ± 0.1	0.7 ± 0.1	90.4 ± 9.3	96.5 ± 6.8	24.1 ± 5.3	43.1 ± 8.3	100	100	86.5 ± 4.7	98 ± 10.2
**50% SeSW**										
***L. p ^#^***	0.44 ± 0.1	0.96 ± 0.1	73.9 ± 8.4	NA	42.5 ± 13.3	NA	93.5 ± 21.7	NA	84 ± 12.2	NA
***A. f ^##^***	0.3 ± 0.1	0.6 ± 0.1	100	100	89.2 ± 14.5	93.2 ± 15.4	100	100	70.5 ± 1.8	95 ± 12.3
***L. p ^##^***	0.31 ± 0.1	0.8 ± 0.1	100	100	41.2 ± 9.9	65.2 ± 8.4	100	100	65.5 ± 3.9	94 ± 13.4

^#^*L. punctata* as a first bioremediating agent; ^##^ *A. filiculoides* or *L. punctata* as the second bioremediating agents.; * Uptake after first 5 days of treatment; ** Total uptake after 10 days of treatment.

**Table 2:** Selenium accumulation in *L. punctata* and *A. filiculoides* biomass.

Wastewater Dilutions	Se, mg/g DW
*L. punctata* (Primary Treatment)	
Control	0.042 ± 0.01
50% *	0.25 ± 0.1
100% *	0.56 ± 0.1
*A. filiculoides* (Secondary Treatment)	
Control	0.02 ± 0.01
50% **	0.18 ± 0.09
100% **	0.42 ± 0.01
*L. punctata* (Secondary Treatment)	
Control	0.01 ± 0.03
50% **	0.16 ± 0.09
100% **	0.38 ± 0.01

Control: Se accumulation at time 0; * Day 5 of primary treatment; ** Day 5 of secondary treatment.

**Table 3:** Supernatant analysis for enzyme activity.

Strains	Starch		Cellulose		*L. punctata*				*A. filiculoides*			
	Amylase, Units min^−1^ mL^−1^		Cellulase, Units min^−1^ mL^−1^		Amylase, Units min^−1^ mL^−1^		Cellulase, Units min^−1^ mL^−1^		Amylase, Units min^−1^ mL^−1^		Cellulase, Units min^−1^ mL^−1^	
	t = 0	t = 72 h	t = 0	t = 72 h	t = 0	t = 72 h	t = 0	t = 72 h	t = 0	t = 72 h	t = 0	t = 72 h
**MAN43**	10.2 ± 3.1	29.3 ± 6.7	2.0 ± 0.7	18.1 ± 4.3	13.4 ± 4.1	33.4 ± 11.2	6.2 ± 2.1	9.0 ± 0.9	10.2 ± 3.4	28.7 ± 8.1	6.3 ± 2.2	18.4 ± 6.3

**Table 4:** Compositional analysis of reduced sugars after acid and enzyme.

Reducing Sugars	Acid Treatment	Enzymatic Treatment
	**Concentration (g/L)**	**Concentration (g/L)**
Glucose	12 ± 0.04	11.80 ± 0.04
Xylose	4.36 ± 0.05	0.924 ± 0.03
Arabinose	0.04 ± 0.02	0

**Table 5:** Soluble metabolites production during hydrogen production by *E. cloacae* DT-1 under decreased partial pressure. Hydrogen productivity and yield efficiency details indicated in detail.

Biomass Treatment	H_2_ Production, (mmol/L)	Volatile Fatty Acid		B/A Ratio	H_2_ Yield *
		Acetic Acid, (g/L)	Butyric Acid, (g/L)		
**Acid Treated Prehydrolysate**	62 ± 0.08	0.712 ± 0.03	0.302 ± 0.03	0.42	2.14 ± 0.04
**Enzymatic Saccharified Hydrolysate**	40 ± 0.05	0.726 ± 0.02	0.302 ± 0.04	0.55	1.8 ± 0.07

B/A ratio: Butyrate/Acetate ratio; * mol H_2_/mol of reduced sugar.

## Supplementary Materials

The following are available online at https://www.mdpi.com/2223-7747/9/4/437/s1, Figure S1: Representation of aquatic plants used for phytoremediation., Figure S2: Experimental design., Figure S3: Changes in concentrations of *C. vulgaris*, light penetration intensity and nutrients by mats of *L. punctata* and *A. filiculoides*., Figure S4: Total carbohydrate yields in *A. filiculoides*, and *L. punctata*., Figure S5: Phylogenetic analysis of thraustochytrid isolate, MAN43., Figure S6: Thraustochytrid cells growth and lipid production on different carbon sources., Table S1: Chemical compositions of SeSW media., Table S2: Hydrogen production from terrestrial and aquatic feedstocks.
