# Supplementary material for: Aquatic Plants, Landoltia punctata, and Azolla filiculoides as Bio-Converters of Wastewater to Biofuel
Source: Plants (Basel). 2020 Apr 1;9(4):437. doi: 10.3390/plants9040437 (PMC7238415; doi:10.3390/plants9040437)
Supplement: Supplementary file 1 [file plants-09-00437-s001.pdf]

Supplementary Materials:

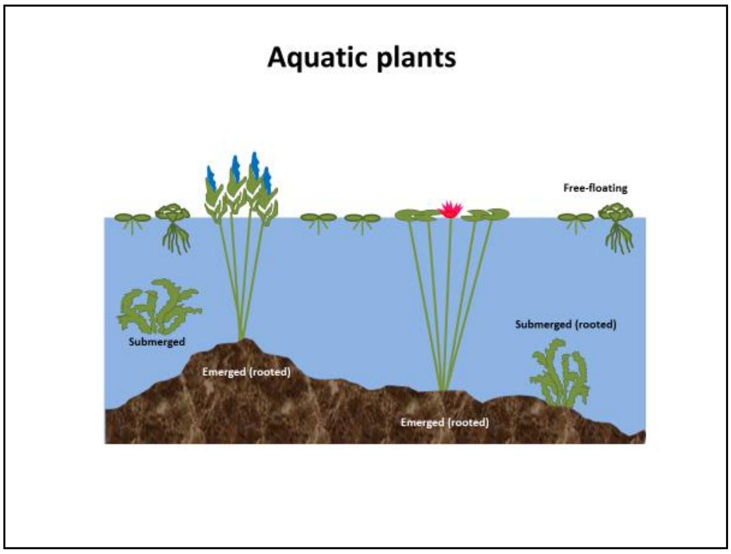

Figure S1. Representation of aquatic plants used for phytoremediation.

Table S1. Chemical composition of SeSW.

Ingredients	Concentration per L
KH_2PO_4	0.585 g
K_2HPO_4	0.218 g
Na_2HPO_4. 7H2O	0.503 g
$NaNO_3$	0.018 g
NH_4Cl	0.060 g
$CaCl_2$	0.0275 g
$MgSO_4$.7H2O	0.0225 g
$Se0_2$	800 µg (600 µg Se)

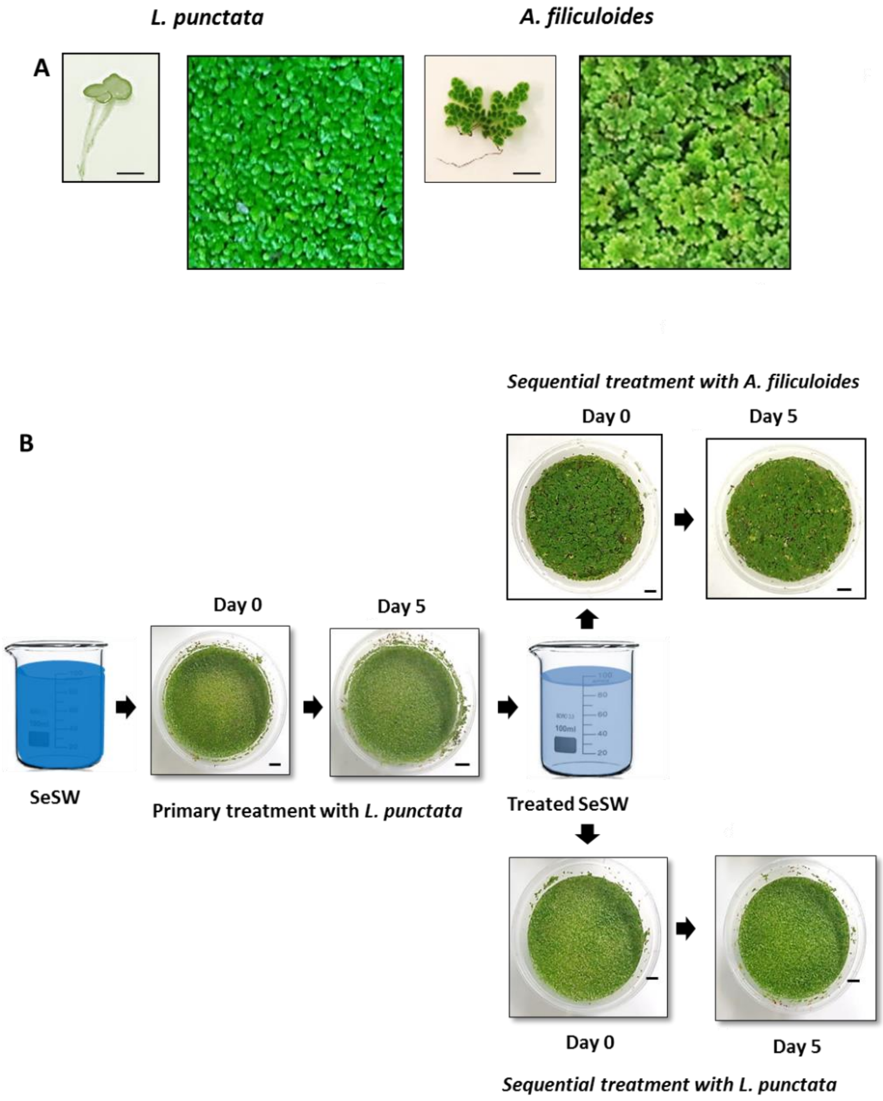

Fig. S2

Experimental design:

A: L. punctata and A. filiculoides plants

B: Experimental design: 100% and 50% SeSW were primarily treated for five days by *L. punctata*. After five days all L. punctata plants were removed, the treated SeSW was filtered, and A. *filiculoides* or *L. punctata* (Control) plants were added followed by an additional five days of treatment. Scale bars: 1cm.

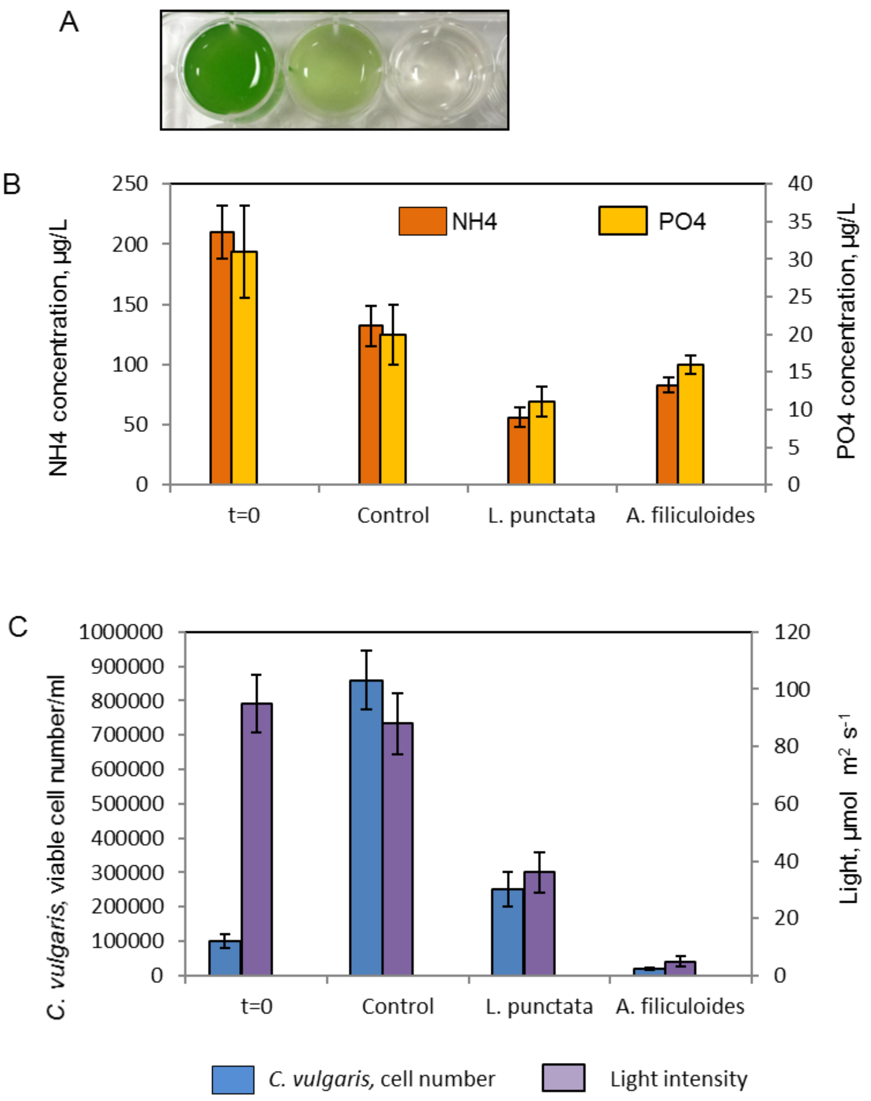

Fig. S3

Changes in concentrations of *C. vulgaris*, light penetration intensity and nutrients by mats of *L. punctata* and *A. filiculoides*

A: Images of *C. vulgaris* cultures grown in control container (top), in containers covered by *L. punctata* (middle) and *A. filiculoides* (bottom).

B: Reduction in concentrations of NH_4 and PO_4 in containers covered by *L. punctata* and *A. filiculoides;*

C: Reduction in concentrations of *C. vulgaris* viable cells and light penetration intensity in containers covered by *L. punctata* and *A. filiculoides.*

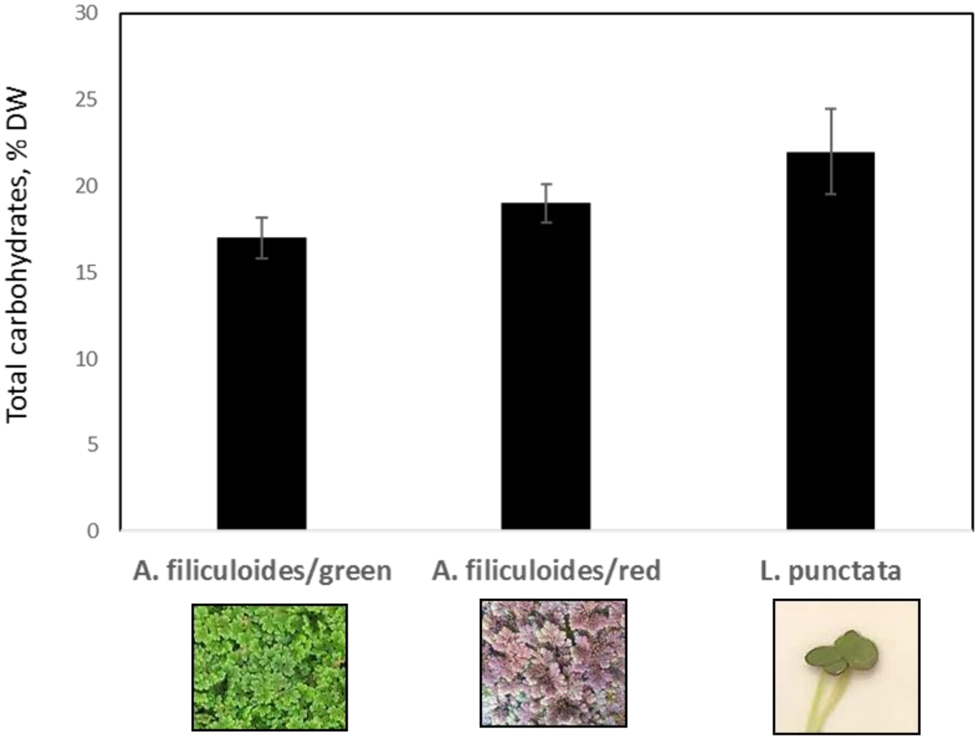

Fig. S4

Figure S4. Total carbohydrate yields in *A. filiculoides,* and *L. punctata.*

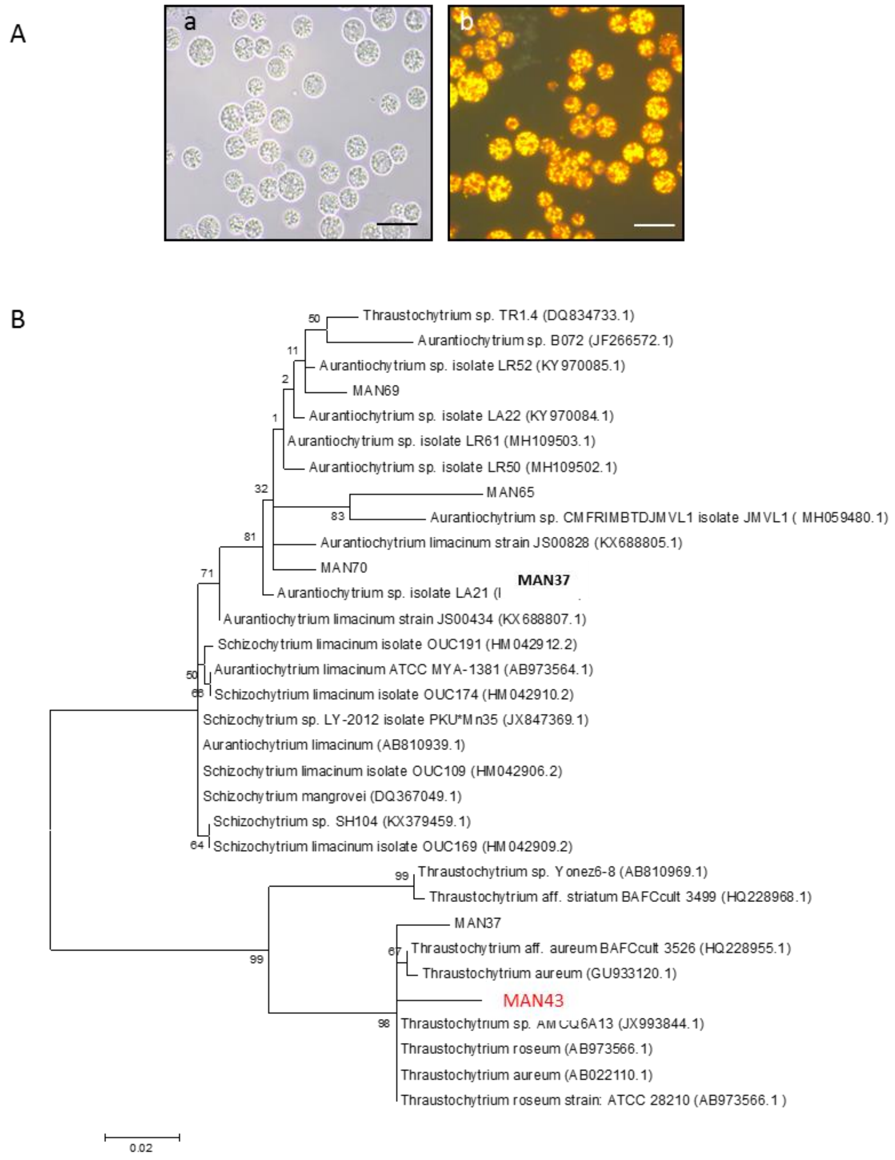

Figure S5

Figure S5. Phylogenetic analysis of thraustochytrid isolate, MAN43.

A: MAN43 cells; a) bright-field microscopy; b) fluorescent microscopy, stained with Nile Red for lipid analysis. Scale bar: 20 µm.

B: Molecular phylogenetic analysis of 18S rRNA sequences by maximum likelihood method showing a close relationship between the isolates and other selected sequences.

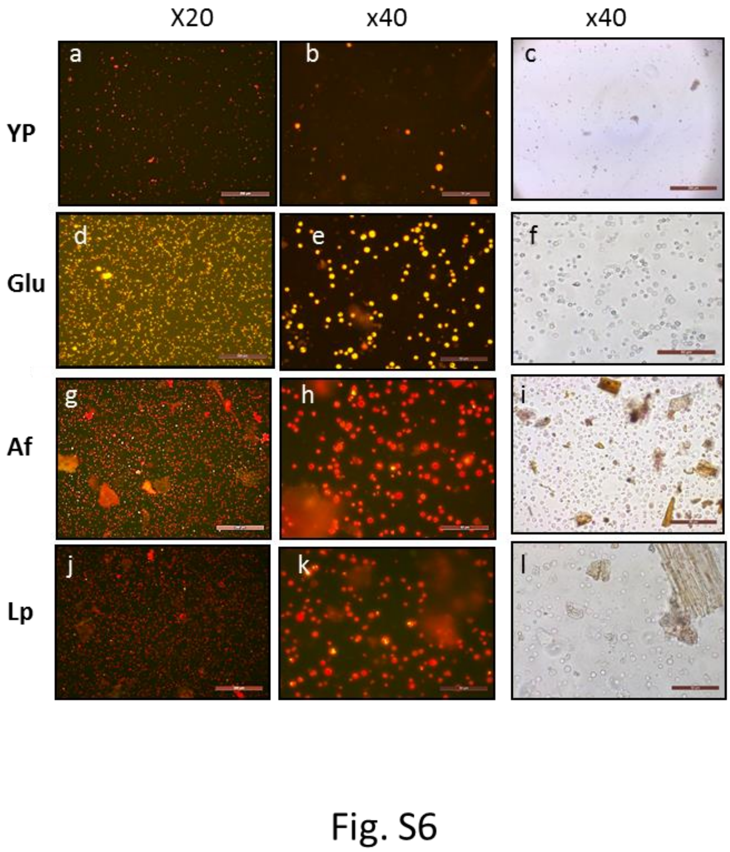

Figure S6. Thraustochytrid cells growth and lipid production on different carbon sources.

Images of MAN643 cells at day three stained with Nile Red grown on YP media (a-c); and YP supplemented with carbon sources. Scales: x20 = 50 µm and x40= 200 µm. Glu: glucose (d-f), Af: *A. filiculoides* (g-i); Lp: *L. punctata* (j-l). Bright field microscopy: c,f,I,l; b) fluorescent microscopy, stained with Nile Red for lipid analysis: a,b,d,e,g,h,j,k.

**Table S2.** Hydrogen production from terrestrial and aquatic feedstocks			
**Feedstock**	**Inoculum**	**Yield**	REF
Cellulose	*Clostridium cellulolyticum*	1.7 mol H2/mol hexose consumed	1
Wheat starch	Anaerobic digester sludge	1.3 mol H2/mol hexose consumed	2
Sugarcane bagasse	*Clostridium butyricum*	1.73 mol H2/mol total sugar	3
Wheat straw	Enrichment culture	2.56 mole H2/mole of hexose	4
Corn stover	Digested sludge	2.84 mole H2/mole of hexose	5
Corn stalks	*Clostridium acetobutylicum*	82 L kg_1	6
Cassava pulp	*Clostridium butyricum, Enterobacter aerogenes*	2.76 mole H2/mole of hexose	7
Wheat starch	Anaerobic sludge	2.84 mole H2/mole of hexose	8
Bagasse	*Clostridium pasteurianum*	0.96 mole H2/mole of hexose	9
Corn stalk waste	Enrichment culture	122 L kg	10
Beet-pulp	Anaerobic sludge	0.79 mole H2/mole of hexose	11
Rice straw	Sewage treatment plant	0.95 mole H2/mole of hexose	12
Duckweed	Anaerobic sludge	45ml/L	13
Spirodela polyrrhiza	Anaerobic sludge	75 mL H2/g	14
L. punctata	*Enterobacter cloacae*	2.14 mol H2/mole of reduced sugar	This study
Azolla filiculoides	*Enterobacter cloacae*	2.43 mol H2/mole of reduced sugar	15

References for Table S2

1. Ren Z, Ward TE, Logan BE, Regan JM: Characterization of the cellulolytic and hydrogen-producing activities of six mesophilic Clostridium species. *J Appl Microbiol.* **2007**, *103*, 2258–2266.
2. Hussy I, Hawkes FR, Dinsdale R, Hawkes DL: Continuous fermentative hydrogen production from a wheat starch co-product by mixed microflora. *Biotechnol Bioeng.* **2003**, *84*, 619–626.
3. Pattra S, Sangyoka S, Boonmee M, Reungsang A: Bio-hydrogen production from the fermentation of sugarcane bagasse hydrolysate by Clostridium butyricum. *Int J Hydrogen Energ.* **2008**, *33*, 5256–5265.
4. Kongjan P, Angelidaki I: Extreme thermophilic biohydrogen production from wheat straw hydrolysate using mixed culture fermentation: Effect of reactor configuration. *Bioresource Technol.* **2010**, *101*, 7789–7796.
5. Datar R, Huang J, Maness P-C, Mohagheghi A, Czernik S, Chornet E: Hydrogen production from the fermentation of corn stover biomass pretreated with a steam-explosion process. *Int J Hydrogen Energ.* **2007**, *32*, 932–939.
6. Ren N, Wang A, Gao L, Xin L, Lee D-J, Su A: Bioaugmented hydrogen production from carboxymethyl cellulose and partially delignified corn stalks using isolated cultures. *Int J Hydrogen Energ.* **2008**, *33*, 5250–5255.
7. Phowan P, Reungsang A, Danvirutai P: Bio-hydrogen Production from Cassava Pulp Hydrolysate using Co-culture of Clostridium butyricum and Enterobacter aerogenes. *Biotechnology.* **2010**, *9*, 348–354.
8. Yokoyama H, Moriya N, Ohmori H, Waki M, Ogino A, Tanaka Y: Community analysis of hydrogen-producing extreme thermophilic anaerobic microflora enriched from cow manure with five substrates. *Appl Microbiol Biot.* **2007**, *77*, 213–222.
9. Chu C-Y, Wu S-Y, Tsai C-Y, Lin C-Y: Kinetics of cotton cellulose hydrolysis using concentrated acid and fermentative hydrogen production from hydrolysate. *Int J Hydrogen Energ.* **2011**, *36*, 8743–8750.

10. Cheng X-Y, Liu C-Z: Fungal pretreatment enhances hydrogen production via thermophilic fermentation of cornstalk. *Appl Energ.* **2012**, 9, 1–6.
11. Ozkan L, Erguder TH, Demirer GN: Effects of pretreatment methods on solubilization of beet-pulp and bio-hydrogen production yield. *Int J Hydrogen Energ.* **2011**, *36*, 382–389.
12. Chang ACC, Tu Y-H, Huang M-H, Lay C-H, Lin C-Y: Hydrogen production by the anaerobic fermentation from acid hydrolyzed rice straw hydrolysate. *Int J Hydrogen Energ.* **2011**, *36*, 14280–14288.
13. Aslan M. Effects of Organic Loading, pH and Temperature on Biohydrogen Production from Duckweed. Energy sources 2015.
14. Xu J, Deshusses. Fermentation of swine wastewater-derived duckweed for biohydrogen production. *Int J Hydrogen Energ.* **2015**, *40*, 7028–7036.
15. Miranda A F,Biswas B, Ramkumar N, Singh R, Kumar J, James A, Roddick F, Lal B, Subudhi S, Bhaskar T Mouradov A. Aquatic plant Azolla as the universal feedstock for biofuel production. *Biotechnol Biofuels* **2016**, *9*, 221

© 2020 by the authors. Submitted for possible open access publication under the terms and conditions of the Creative Commons Attribution (CC BY) license (http://creativecommons.org/licenses/by/4.0/).
